# Isolation, Identification of *Serpula himantioides* from Dingtao M2 Tomb and Its Wood Degradation Characteristics

**DOI:** 10.3390/ijms27146422

**Published:** 2026-07-19

**Authors:** Yu Wang, Cen Wang, Lilong Hou, Zeao Wang, Zhiqian Guan, Jiao Pan

**Affiliations:** 1 Key Laboratory of Archaeomaterials and Conservation, Ministry of Education, University of Science and Technology Beijing, Beijing 100083, China; 2 Institute for Cultural Heritage and History of Science & Technology, University of Science and Technology Beijing, Beijing 100083, China; 3 College of Life Sciences, Nankai University, Tianjin 300071, China

**Keywords:** *Serpula himantioides*, Dingtao M2 tomb, wood degradation, cellulase, genome sequencing

## Abstract

The Dingtao M2 Tomb, the largest, highest-specification, and best-preserved “Huangchangticou” tomb currently discovered in China, is of great significance for cultural relic conservation. During its dismantling and protection, extensive white filamentous fungal contamination was observed on the surface of sand-buried wood components. To clarify the dominant fungal species and its impact on wood cultural relics, the dominant fungus was isolated and purified from contaminated wood samples, and identified as *Serpula himantioides* (designated as DTW) via molecular and morphological methods. Systematic studies were conducted on DTW’s wood degradation capacity, cellulase activities, regulatory effects of Fe^3+^ and Ca^2+^ on cellulase activities, whole-genome characteristics, and sensitivity to fungistatic agents. The results indicated that DTW exhibited strong wood degradation ability: after 60 days of inoculation, the maximum force, elongation at break, and tensile strength of wood chips decreased significantly, by 88.0%, 54.6%, and 88.6%, respectively, compared with the control group. The cultural relic microenvironment colonized by strain DTW is rich in Fe^3+^ and Ca^2+^. Both ions can significantly suppress the activity and specific activity of cellulase from strain DTW at elevated concentrations. Whole-genome sequencing revealed that DTW had a genome size of 68,519,434 bp with a Guanine–Cytosine (GC) content of 44.01% and 18,373 genes; Carbohydrate-Active Enzyme (CAZy) and Cell Wall-Degrading Enzyme (CWDE) database annotations confirmed its strong plant cell wall degradation ability. Additionally, DTW was sensitive to screened green fungistatic agents, which effectively inhibited its growth. This study clarifies the species and wood degradation mechanism of the dominant fungus on Dingtao M2 Tomb’s sand-buried wood, providing theoretical and technical support for the protection of the tomb’s wood cultural relics.

## 1. Introduction

Wooden artifacts are irreplaceable organic cultural relics that preserve direct physical evidence of ancient civilizations [1,2,3]. Long-term underground burial under complex geochemical conditions inevitably induces multiple forms of damage to archaeological wood, and biodeterioration mediated by microorganisms has attracted mounting research attention within heritage conservation over recent decades [4,5,6,7,8,9,10,11].

Among all wood-degrading microorganisms, fungi represent the most abundant and functionally powerful decomposers of lignocellulosic substrates [12,13,14,15]. Unlike single-celled prokaryotes, their extensive filamentous hyphae enable efficient spread and deep penetration into wood matrices. Wood-rotting fungi are classified into three canonical functional groups based on their degradation patterns toward plant biopolymers: white-rot fungi fully decompose cellulose, hemicellulose, and lignin; brown-rot fungi break down polysaccharides while leaving partially modified lignin behind; soft rot, predominantly produced by ascomycetes, targets cellulose and hemicellulose and generates distinctive conical and spongy microcavities inside wood tissues [16,17,18,19,20].

The Dingtao M2 Tomb in Heze’s Dingtao District dates back to the late Western Han Dynasty (202 BC–8 AD), with a burial history spanning more than 2000 years. This grand Huangchangticou mausoleum is the largest, highest-ranked and best-preserved tomb of its kind excavated in China to date, as well as the biggest sealed tomb among three burial complexes at the same archaeological site [12,15]. Its excavation marks a milestone in Chinese archaeology. Beyond reflecting the brilliant civilization of ancient Dingtao, the tomb physically validates the prevalence of elaborate elite burial customs in the Western Han, whose architectural layouts and burial styles differ markedly from earlier dynasties. Accordingly, the site carries unique historical value for studying Han Dynasty social structures, burial philosophies, cultural ideologies, and perceptions of life and death [21,22].

Large quantities of wooden structural components have been unearthed from the tomb, which is currently undergoing systematic disassembly and on-site conservation. Conservation staff have adopted multiple dehydration techniques for recovered wooden relics; specifically, an in-tank sand-burial method was implemented on-site to treat the tomb’s cover planks for dehydration stabilization, a customized operation exclusive to this heritage protection project. When excavated, all wooden relics remained heavily waterlogged after millennia of burial, already afflicted with inherent deterioration such as cracking and salt efflorescence. Worse, widespread white filamentous fungal contamination has recently appeared on the surfaces of sand-buried wood pieces. Protecting large-scale waterlogged wooden heritage remains a worldwide technical bottleneck in conservation practice. As the wooden components of Dingtao M2 Tomb carry irrecoverable historical information, uncontrolled fungal decay poses an irreversible threat to the integrity of these precious relics.

Faced with this urgent on-site conservation challenge, the present study takes the sand-buried wooden relics of Dingtao M2 Tomb as the research subject. We isolate and identify the dominant pathogenic fungus contaminating the wood, systematically characterize its wood degradation potential and cellulase metabolic properties, and explore how environmental Fe^3+^ and Ca^2+^ regulate its enzymatic activity. Eco-friendly fungistatic agents are also screened to develop feasible inhibition strategies. This work clarifies the fungal biodeterioration mechanism targeting sand-buried Han wooden relics, provides solid theoretical support and operable technical schemes for the long-term preservation of the Dingtao tomb, and offers a valuable reference for microbial prevention and conservation of analogous waterlogged wooden cultural relics around the world.

## 2. Results and Discussion

### 2.1. Isolation, Purification and Molecular Identification of the Dominant Fungus

**Figure 1:**
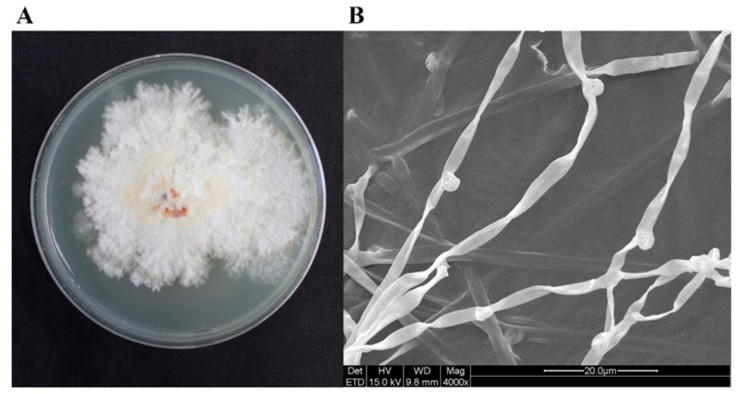
Growth morphology of *S. himantioides* DTW. (**A**) PDA medium, incubated at 25 °C for 20 days; (**B**) SEM observations.

Molecular identification results confirmed that the dominant fungus on the wood cultural relics was *Serpula himantioides.* Belonging to Basidiomycota, *S. himantioides* possesses well-developed hyphae with septa. The collected microbial samples were inoculated onto PDA plates and cultured at a constant temperature of 25 °C for 7 days. After repeated isolation and purification by the streak plate method, a pure culture of *S. himantioides* was finally obtained, which was designated as DTW and stored at 4 °C for subsequent experimental use. Single-colony culture experiments were carried out to observe the growth characteristics of DTW. The hyphae of *S. himantioides* DTW grown on PDA medium were initially white, fluffy and cotton-like in texture; with the extension of culture time, the hyphae gradually turned light yellow and then deep yellow, and finally turned brown in the late growth stage, with the colony edge remaining slightly white (Figure 1A). To further clarify its morphological characteristics, SEM was used to observe the growth morphology of DTW. The results showed that DTW did not produce spores on PDA medium, and its hyphae were straight or slightly curved, with obvious septa; notably, some hyphae could intertwine and form unique ring-shaped structures, which might be related to its growth and colonization ability (Figure 1B).

Molecular and morphological identification confirmed that the dominant fungus isolated from the sand-buried wood of Dingtao M2 Tomb was *S. himantioides* of Basidiomycota. Consistent with previous reports, their hyphae can intertwine to form unique ring-shaped structures [23,24,25]. This ring-shaped structure may be an adaptive trait of strain DTW, facilitating the adhesion, nutrient transport and colonization of hyphae on the surface of sand-buried wood, endowing it with a survival advantage in the relatively dry and sand-rich microenvironment of the wood dehydration system in the tomb [26,27,28]. Fungi of the genus *Serpula* are globally distributed wood-decaying fungi, commonly found in forests and wooden structures, with records in Asia, Europe, North America, Australia and other regions [29,30,31]. Fungi of this genus can infect a variety of coniferous and broad-leaved trees, including *Larix kaempferi*, *Chamaecyparis pisifera*, *Pseudotsuga menziesii*, etc. [32,33,34]. Notably, *S. himantioides* is mainly reported in temperate and cold forest ecosystems and is relatively rare in wooden buildings [35]. This study is the first to isolate and identify *S. himantioides* from sand-buried wooden cultural relics, expanding the known ecological niche of this species to archaeological wood habitats.

### 2.2. Degradation of Wood by S. himantioides DTW

**Figure 2:**
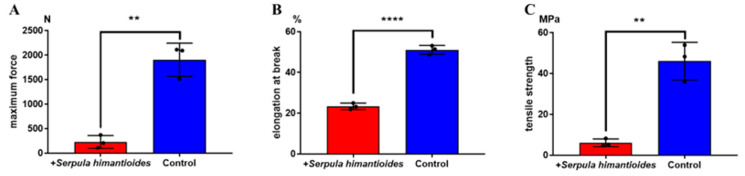
Effect of *S. himantioides* DTW on wood mechanical properties (60 days). (**A**) Maximum force; (**B**) elongation at break; (**C**) tensile strength. (** *p* < 0.01; **** *p* < 0.0001).

To evaluate the wood degradation ability of *S. himantioides* DTW, a universal mechanical testing machine was used to systematically determine three key mechanical properties of wood samples after degradation treatment (Section 3.4), including maximum force, elongation at break, and tensile strength. The experimental results showed that the three mechanical indexes of wood in the experimental group were significantly reduced compared with the control group after 60 days of degradation by *S. himantioides* DTW, indicating that DTW had a strong ability to degrade wood. Specifically, the average maximum force of the experimental group was only 229 N, while that of the control group reached 1908 N, which was about 8.3 times higher than that of the experimental group (Figure 2A). In terms of elongation at break, the average value of the experimental group was 23.36%, which was significantly lower than 51.44% from the control group, suggesting that the toughness of wood was greatly weakened after degradation (Figure 2B). For tensile strength, the average value of the experimental group was 5.24 MPa, whereas that of the control group was 46.12 MPa, showing a significant decrease of nearly 89% in the experimental group compared with the control group (Figure 2C).

The degradation of wood by fungi is essentially a process driven by the synergistic action of lignocellulose-degrading enzymes, which ultimately leads to the destruction of the chemical structure and mechanical properties of wood [36,37,38,39,40]. Mechanical property tests in this study showed that strain DTW has strong wood degradation ability. The significant decrease in maximum force and tensile strength indicates that strain DTW severely damages the load-bearing capacity of wood, while the decrease in elongation at break reflects the loss of wood toughness [41,42,43]. These changes pose a serious threat to the structural stability of the wooden components of Dingtao M2 Tomb. Considering that the wood of the tomb has suffered from cracking, salt efflorescence, water immersion and other damages after more than 2000 years of burial, the degradation by strain DTW may accelerate the fragmentation and collapse of wood, leading to the loss of precious historical information carried by the cultural relics. This highlights the urgency of implementing targeted microbial control measures to protect these precious wooden cultural relics.

### 2.3. Enzymatic Characteristics of Cellulase Produced by Strain DTW

**Figure 3:**
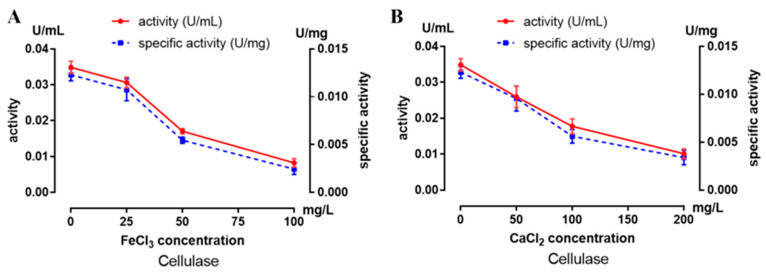
Effects of different concentrations of Fe^3+^ and Ca^2+^ on cellulase activity and specific activity. (**A**) Fe^3+^; (**B**) Ca^2+^.

As a brown-rot fungus, strain DTW was assayed for cellulase activity and specific activity. On day 21 of cultivation, its cellulase activity was 0.0892 U/mL, with a specific activity of 0.0252 U/mg. XRF analysis was performed to determine the elemental composition of the wood samples. The results showed that the wood samples contained large amounts of iron (Fe) and calcium (Ca), with their average relative contents reaching 58.749% and 16.613%, respectively. In addition, trace amounts of other elements such as silicon (Si), sulfur (S), and aluminum (Al) were also detected in the wood samples (Table S1). Based on these findings, it was speculated that the growth environment of DTW on the surface of sand-buried wood might contain relatively high levels of Fe^3+^ and Ca^2+^. Therefore, experiments (Section 3.7) were conducted to investigate the effects of these two metal ions on the cellulase activity and specific activity of DTW by adjusting the concentrations of Fe^3+^ and Ca^2+^. The results showed that cellulase activity remained stable in PD medium supplemented with 25 mg/L Fe^3+^. By contrast, cellulase activity decreased markedly when the Fe^3+^ concentration increased to 50 mg/L and to 100 mg/L (Figure 3A). Similarly, Ca^2+^ at concentrations of 50, 100, and 200 mg/L all caused a significant reduction in cellulase activity (Figure 3B).

Lignin and cellulose are the core structural components of wood, and their degradation depends on the synergistic action of specific extracellular enzymes secreted by fungi. This study confirmed that strain DTW can secrete cellulase, which is consistent with the enzyme secretion profile of typical brown-rot fungi. It is known that metal ions such as Fe^3+^ and Ca^2+^ can regulate the activity of cellulase in wood-decaying fungi as cofactors, inhibitors, or activators depending on their concentrations [44,45,46,47]. This study found that Fe^3+^ and Ca^2+^ have significant regulatory effects on the cellulase of strain DTW. It is known that metal ions can stabilize the enzyme structure, but excessive metal ions may interfere with the binding of enzymes to their substrates or damage the fungal cell membrane, thereby inhibiting enzyme secretion [46]. This study only verified the phenotypic inhibitory effects of high Fe^3+^ and Ca^2+^ concentrations on cellulase activity in strain DTW. The intrinsic mechanism underlying metal ion-mediated cellulase regulation and the adaptive strategy of strain DTW toward metal-enriched wooden relics remains elusive. Specifically, it is unclear how this strain successfully colonizes and decays wooden cultural relics despite suppressed cellulase activity under high-metal conditions. Therefore, further investigations are essential to elucidate the molecular mechanisms by which metal ions modulate cellulase function in strain DTW, which will deepen our understanding of fungal deterioration mechanisms of ancient wooden relics.

### 2.4. Whole-Genome Sequencing Analysis of DTW

**Figure 4:**
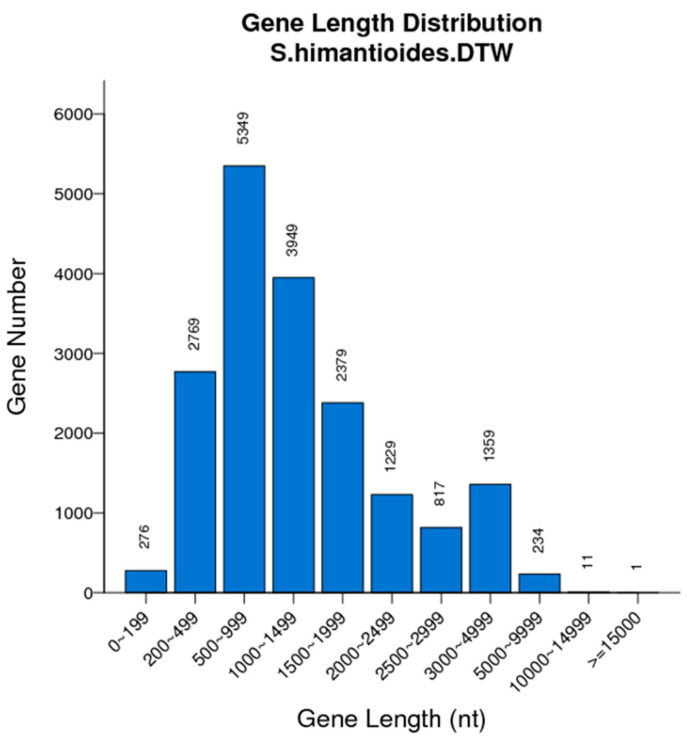
Gene length distribution.

The whole-genome sequencing results showed that the genome size of *Serpula himantioides* DTW was 68,519,434 bp with a GC content of 44.01%. A total of 18,373 genes were identified, with an average gene sequence length of 1792 bp (Figure 4). The total length of all gene sequences was 32,924,400 bp, accounting for 48.05% of the total genome size. Additionally, the total length of CDS (Coding Sequence) was 25,614,278 bp, accounting for 37.38% of the whole genome. The DTW genome contained 102,480 exons and 84,107 introns. Whole-genome sequencing also analyzed the information of five types of non-coding RNAs (ncRNAs) in DTW, with detailed data presented in Table S2. Repetitive sequences in the DTW genome, including DNA transposons, tandem repeats, and other transposable elements, were predicted to account for 43.75% of the total genome.

**Figure 5:**
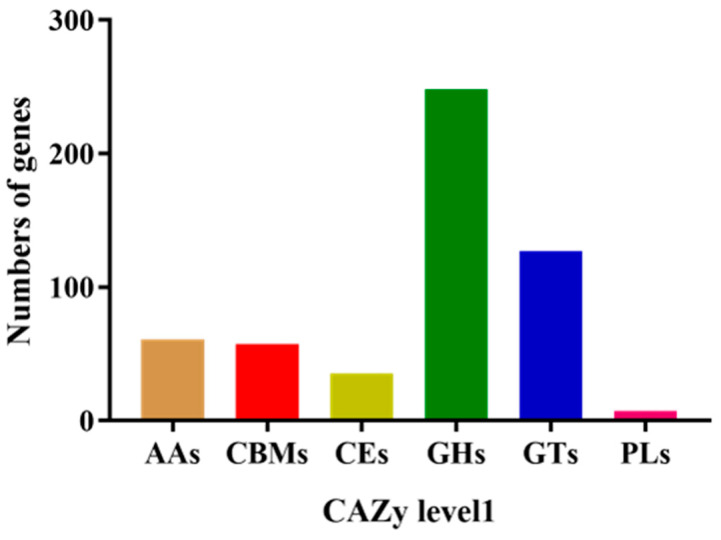
Functional annotation distribution of CAZy database Level 1 in the whole-genome analysis of *S. himantioides* DTW.

**Figure 6:**
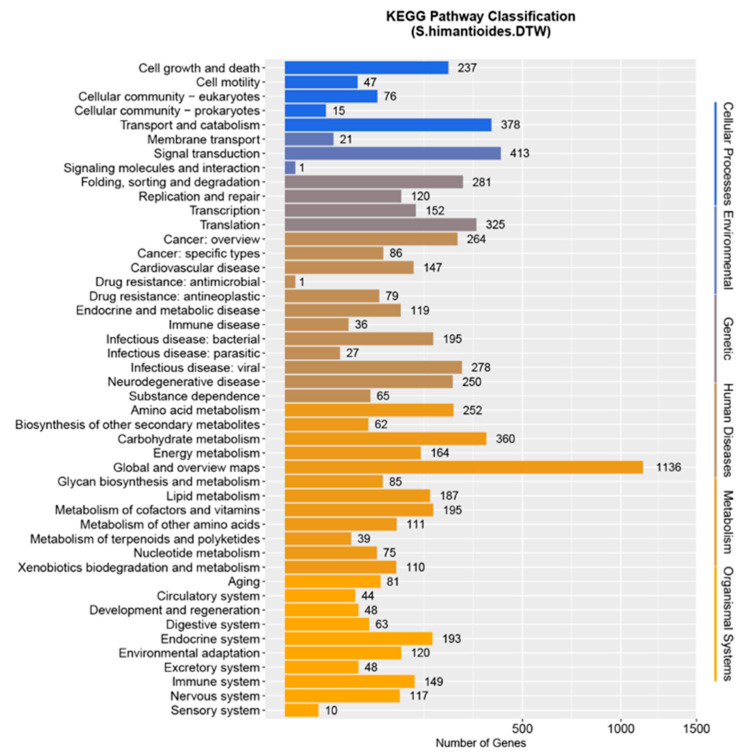
Functional annotation distribution of secondary classification of KEGG database in the whole-genome analysis of *S. himantioides* DTW.

After obtaining the whole-genome sequence of DTW, gene comparison and annotation were performed in multiple databases to explore gene functions. A total of 14,772 genes were annotated across 16 databases, accounting for 80.4% of the total genes. The number and proportion of annotated genes in each database are shown in Table S3. Further analysis was focused on the annotation results from CAZy, CWDE, and KEGG databases. In the CAZy database Level 1 annotation, glycoside hydrolases (GHs) were the most abundant annotated genes, while polysaccharide lyases (PLs) were the least (Figure 5). A total of 46 genes of DTW were annotated in the CWDE (Plant Cell Wall-Degrading Enzyme) database, accounting for 0.25% of the total genes, indicating that DTW possesses strong plant cell wall degrading ability. In the KEGG database Level 2 annotation, carbohydrate metabolism accounted for a relatively large proportion (Figure 6).

Whole-genome sequencing results showed that the genome size of strain DTW is 68,519,434 bp, with a GC content of 44.01%, and a total of 18,373 genes were annotated. In multiple databases, the proportion of annotated genes is as high as 80.4%, indicating the completeness and reliability of the genome sequence, laying a solid foundation for exploring the functional potential of strain DTW. CAZy database annotation showed that glycoside hydrolases are the most abundant genes, which are closely related to the hydrolysis of polysaccharides such as cellulose and hemicellulose. In addition, 46 genes were annotated in the Plant Cell Wall-Degrading Enzyme database, confirming that strain DTW has strong plant cell wall degradation ability. KEGG database annotation showed that the proportion of genes involved in carbohydrate metabolism is relatively high, further supporting the ability of strain DTW to use woody carbohydrates as energy sources. This comprehensive genomic analysis not only confirms the wood degradation potential of strain DTW at the molecular level, but also provides candidate genes for further studying the regulatory mechanisms of enzyme secretion and metal ion adaptation in *S. himantioides*.

### 2.5. Sensitivity of S. himantioides DTW to Different Fungistatic Agents

**Figure 7:**
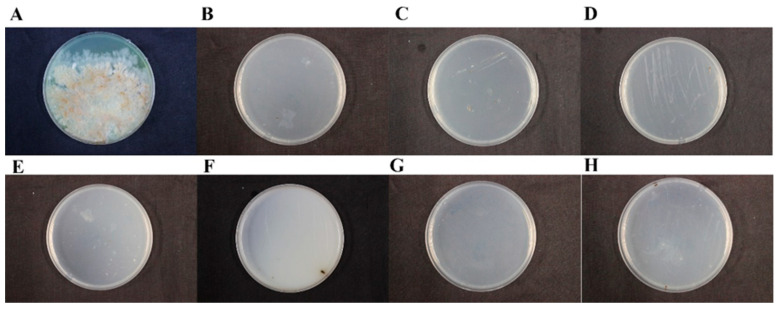
Inhibition effects of different fungistatic agents on *S. himantioides* DTW, incubated at 25 °C for 30 days. (**A**) Negative control; (**B**) 0.5% K100; (**C**) 0.05% BC01; (**D**) 0.3% BC01; (**E**) 0.5% BC08; (**F**) 3.5% BC08; (**G**) 0.025% BC14; (**H**) 0.5% BC14.

In our previous laboratory experiments, we screened several eco-friendly fungistatic agents with remarkable antifungal activity, including commercial isothiazolinone biocides K100, BC01, and BC14. On this basis, the sensitivity of *S. himantioides* DTW to these screened fungistatic agents was further determined. The experimental results indicated that all the selected fungistatic agents could effectively inhibit the growth of DTW on the culture medium (Figure 7), suggesting that these fungistatic agents have great application potential in the field of cultural relic protection.

The protection of wooden cultural relics requires fungistatic agents that are both effective and environmentally friendly to avoid damaging the cultural relics or causing ecological pollution. The fungistatic agents used in the experiment have been applied to other wooden cultural relics [13]. This study found that all the screened green fungistatic agents can effectively inhibit the growth of strain DTW, which indicates that these green fungistatic agents have great application potential in the microbial control of the wooden cultural relics of Dingtao M2 Tomb.

The current study only completed 30-day static fungistatic screening tests under laboratory pure culture conditions, which verifies the immediate inhibitory efficacy of 0.5% K100, 0.05% BC01, 0.3% BC01, 0.5% BC08, 3.5% BC08, 0.025% BC14 and 0.5% BC14 against *S. himantioides* DTW. However, long-term domestication experiments on strain resistance evolution were not carried out in this work, which we clearly state as a limitation of the present research. We explain that the inhibitory effect is not simply a transient response to novel chemical stress; these green fungistatic agents target key physiological processes of brown rot fungi including cell membrane permeability, extracellular cellulase secretion, and mycelial extension. Even under continuous low-concentration exposure, the agents sustain growth suppression, rather than only exerting short-term acute inhibition. We propose future research directions: continuous subculture domestication experiments to explore whether DTW will produce adaptive tolerance after long-term fungicide exposure, and multi-round concentration gradient screening to evaluate resistance risk, which will further support the long-term on-site application of these biocides for cultural relic protection.

## 3. Materials and Methods

### 3.1. Microbial Contamination Investigation and Sample Collection

**Figure 8:**
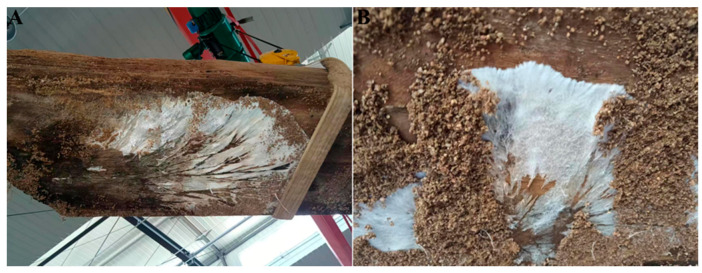
Microbial contamination on the wood of Dingtao M2 tomb. (**A**) Overall appearance; (**B**) local appearance.

The Dingtao M2 Tomb is currently undergoing systematic disassembly and on-site conservation treatment. The unearthed wooden structural components were continuously saturated by underground groundwater for over two thousand years during burial. To prevent rapid shrinkage and severe cracking after excavation, multiple targeted dehydration conservation approaches were deployed on recovered wooden relics, among which the in-tank sand-burial method was specially adopted for dehydration stabilization of the tomb’s cover planks. During regular routine inspections by professional conservators from the Dingtao M2 Tomb Conservation Institution, extensive white filamentous fungal colonies were discovered covering the surfaces of sand-buried wooden elements (Figure 8). Motivated by this on-site fungal contamination risk, we collected microbial and wood samples from visibly deteriorated areas. All collected samples were transported back to the laboratory; microbial samples were subjected to isolation and purification culture to obtain pure fungal strains and subsequent whole-genome sequencing, while the collected wood samples were processed with X-ray Fluorescence (XRF) elemental composition analysis to characterize the elemental background of the contaminated relic wood.

### 3.2. Isolation, Purification and Molecular Identification of Fungi

Fungal culture was performed using Potato Dextrose Agar (PDA) medium, and the inoculated medium was incubated at 25 °C; isolation and purification were carried out according to the morphological characteristics of fungal colonies, and pure cultures were obtained after 1–3 rounds of isolation, then inoculated onto PDA slant solid medium and stored at 4 °C for subsequent use. The species of the strain was determined by molecular identification using the T5 Direct PCR Kit (Tsingke, Beijing, China) for PCR amplification of the purified fungi: a small amount of hyphae was taken and placed into a PCR tube, 50 μL of Lysis Buffer A was added to the tube, incubated at 95 °C for 10 min, centrifuged briefly, and 2 μL of the supernatant was taken as the template for the PCR reaction; the PCR reaction system was prepared with 2 μL each of upstream primer ITS1(5′-TCCGTAGGTGAACCTGCGG-3′) and downstream primer ITS4 (5′-CCTCCGCTTATTGATATGC-3′), 2 μL of template, 25 μL of MIX, and filled to 50 μL with ddH_2_O, followed by setting the PCR reaction program at 98 °C for 3 min, 31 cycles of 98 °C for 10 s, 57 °C for 10 s, 72 °C for 50 s, 72 °C for 3 min, and holding at 4 °C; the PCR product was detected by electrophoresis with a band size of approximately 550 bp, then sent to a company for sequencing, and the sequencing results were aligned in the NCBI database to determine the species of the strain.

### 3.3. Scanning Electron Microscope (SEM) Observation

Fungi growing on PDA medium were adhered using carbon conductive tape and then dried in a desiccator. After drying, the samples were attached to the SEM sample stage. Gold sputtering was performed at a current of 24 mA for 300 s, followed by SEM observation and image recording. The measurement conditions were set as follows: EHT: 15.0 kV, WD: 9.8 mm, Mag: 4K×.

### 3.4. The Influence of Fungi on the Mechanical Properties of Wood

Firstly, the tested fungi were evenly spread on PDA medium and incubated at 25 °C for 15 days. Wood samples were collected from well-preserved relic timber of the Dingtao M2 Tomb, which had been buried underground for more than 2000 years, and cut into chips of uniform size (0.5 cm × 0.2 cm × 3 cm). These wood chips were sterilized by high-temperature and high-pressure treatment at 121 °C for 30 min, with the sterilization process repeated twice, followed by drying. For the experimental group, wood chips were gently placed on the surface of PDA medium pre-inoculated with the tested fungi; in contrast, the control group was treated by placing identical wood chips on the surface of sterile blank PDA medium without any fungal inoculation. The only variable separating the two groups was the presence of the target fungus in the experimental group. Both groups were incubated at 25 °C for 60 days before the wood chips were taken out for subsequent tests. The mechanical properties of the wood chips were measured using a universal mechanical testing machine, with the gauge length set at 20 mm and the testing speed at 10 mm/min. Three key mechanical parameters were evaluated, and their definitions are as follows: Maximum force refers to the maximum force that the specimen can withstand after the yield stage; for materials without an obvious yield stage (exhibiting continuous yield), it is the maximum force recorded during the entire test process, and a smaller maximum force indicates more severe corrosion of the wood chips. Tensile strength refers to the stress at which a material undergoes maximum uniform plastic deformation; a lower tensile strength implies more severe wood chip corrosion. Elongation at break is usually defined as the ratio of the displacement of the sample at fracture to its original length; a smaller elongation at break indicates more severe corrosion of the wood chips. All three parameters were calculated from the stress–strain curve obtained during the stretching test. Comparisons between the two groups were performed using an unpaired *t*-test, with significance levels defined as ** *p* < 0.01 and **** *p* < 0.0001.

### 3.5. Activity and Specific Activity Detection of Cellulose

To quantitatively assess the extracellular enzyme secretion profile of the test fungus, the activity of cellulase in the fungal culture supernatant was determined using commercial test kits, following the manufacturers’ instructions. Specifically, the protein content in the supernatant was quantified using BCA Protein Assay Kit (PC0020, Solarbio, Beijing, China). Meanwhile, the cellulase activity was analyzed using Cellulase Assay Kit (AK196, Bioss, Beijing, China). In addition to the absolute enzyme activity, the specific activity, defined as the enzyme activity per unit of protein concentration, was calculated to accurately reflect the synthetic and secretory capacities of the fungal strain under different experimental conditions.

### 3.6. X-Ray Fluorescence (XRF) Analysis

The relative contents of various elements in the wood were determined using an X-ray Fluorescence spectrometer. The wood blocks collected from the Dingtao Tomb were crushed by a grinder, dried in a well-ventilated place, and then sieved through an 80-mesh sieve. The sieved wood powder was placed into the test circle inside the instrument and pressed into a compact flat surface, after which the element contents in the wood powder were measured by XRF. The operating parameters of the XRF spectrometer were set as follows: tube voltage of 50 kV, output current of 4 mA, and rated power of 200 W. XRF can realize qualitative and quantitative detection of elements by measuring the characteristic fluorescence wavelength and intensity emitted by the sample after being irradiated with X-rays.

### 3.7. Setting of Different Cultivation Conditions

Calcium chloride (CaCl_2_) and ferrous sulfate (FeSO_4_) were used to set different concentrations of Ca^2+^ and Fe^2+^, respectively, to determine the effects of these metal ion concentrations on the activity of cellulose. The experiment was conducted based on PD liquid medium, with the concentration gradients of Fe^2+^ set as 0 mg/L, 25 mg/L, 50 mg/L, and 100 mg/L, and the concentration gradients of Ca^2+^ set as 0 mg/L, 50 mg/L, 100 mg/L, and 200 mg/L. An equal amount of the fungus was inoculated into each medium, and all groups were cultured for the same duration before the enzyme activity and specific enzyme activity were measured. Statistical analyses were performed using GraphPad Prism software (version 10.1.2). Differences in enzyme activity and specific activity among different CaCl_2_ and FeSO_4_ concentration groups were assessed by one-way analysis of variance (ANOVA).

### 3.8. Genome Sequencing Analysis

To obtain high-quality genomic DNA for whole-genome sequencing, the fungus strain was first cultured in PD liquid medium at a constant temperature of 25 °C for 20 days to ensure sufficient cell proliferation. After the cultivation period, the entire culture system (including fungal mycelia and culture medium) was transferred into 50 mL sterile centrifuge tubes. The tubes were then centrifuged at 4000 rpm for 15 min at 4 °C to separate and collect the fungal mycelial pellets. The collected mycelia were gently rinsed with sterile deionized water to remove residual medium components and impurities, followed by a second centrifugation under the same conditions to further purify the mycelial pellets. To preserve the integrity of genomic DNA and prevent degradation, the purified mycelia were quickly frozen in liquid nitrogen and immediately sent to BGI Genomics (Shenzhen, China) for whole-genome sequencing analysis, which included DNA extraction, library construction, and high-throughput sequencing processes.

### 3.9. Fungistatic Experiment

To inhibit the growth of pathogenic fungi isolated from the sand-buried wood, a fungistatic experiment was conducted to screen for effective and environmentally friendly fungistatic agents. Specifically, different concentrations of the tested fungistatic agents were added to PDA medium prior to its solidification, ensuring uniform distribution of the agents in the medium. After the medium solidified, a sterile cotton swab was used to evenly spread the tested fungal suspension onto the surface of the PDA medium. All inoculated plates were then placed in a constant-temperature incubator at 25 °C and cultured for 30 days. During and after the cultivation period, the growth status of the fungi on each plate was observed and recorded. The main components of the fungistatic agents used in this experiment are detailed in Table S4.

## 4. Conclusions

This study isolated and identified *Serpula himantioides* DTW, the dominant fungus responsible for severe biodeterioration of sand-buried wooden relics from Dingtao M2 Tomb. A series of morphological, mechanical, enzymatic, elemental, genomic, and fungistatic experiments systematically clarified its strong wood degradation capacity, the regulatory effects of environmental Fe^3+^ and Ca^2+^ on its cellulase activity, and the genetic basis supporting its lignocellulose decomposition. The eco-friendly isothiazolinone biocides, including K100, BC01 and BC14, screened in this work can effectively inhibit the growth of DTW, supplying safe antifungal materials for on-site cultural relic conservation.

Fungal decay brings irreversible damage to waterlogged archaeological wood worldwide, while systematic research on wood-rotting fungi from different ancient tomb sites is still limited. This study is the first to document *S. himantioides* isolated from sand-dehydrated wooden artifacts, which highlights the necessity of carrying out more similar investigations on wooden relics from diverse burial environments. Relevant parallel studies can establish a complete database of tomb wood pathogenic fungi and supplement the theoretical system of heritage biodeterioration; meanwhile, more cross-scenario trials are required to verify the universal field applicability of green fungistatic agents and form standardized conservation technical specifications. Future research will combine transcriptomics and proteomics to uncover the molecular mechanism enabling DTW to colonize wood under high Fe^3+^ and Ca^2+^ stress with restrained cellulase activity. Long-term subculture domestication tests will be conducted to evaluate the risk of fungal resistance against isothiazolinone biocides and optimize compound bacteriostatic formulas. We will also construct simulated sand-buried microenvironments, to test the long-term antifungal durability of these agents on authentic ancient wood under complex in situ conditions, and further explore co-degradation effects of mixed fungal flora to develop targeted integrated microbial prevention strategies for real tomb environments.

Overall, this research provides solid theoretical support and practical green antifungal approaches for the protection of wooden relics from Dingtao M2 Tomb. Continued multi-dimensional trials focusing on decay fungi of archaeological wood will promote the development of low-harm microbial control technology for waterlogged wooden cultural heritage.

## Data Availability

The raw sequencing data can be accessed at the NCBI Genome under the study accession number PRJNA1451086.

## Supplementary Materials

The following supporting information can be downloaded at: https://www.mdpi.com/article/10.3390/ijms27146422/s1.
