# Supplementary material for: Isolation, Identification of Serpula himantioides from Dingtao M2 Tomb and Its Wood Degradation Characteristics
Source: Int J Mol Sci. 2026 Jul 19;27(14):6422. doi: 10.3390/ijms27146422 (PMC13410424; doi:10.3390/ijms27146422)
Supplement: Supplementary file 1 [file ijms-27-06422-s001.zip › ijms-4399546-supplementary.pdf]

Table S1. X-ray fluorescence detection data of sand-buried wood

	Wood.1 (%)	Wood.2 (%)	Wood.3 (%)	Wood.4 (%)
Fe	56.9302	56.3074	65.808	55.9499
Ca	17.9204	13.477	15.4455	19.6074
Si	9.4508	11.308	4.2369	7.1535
S	5.0715	7.7802	6.7268	7.0875
Al	2.0156	2.3261	1.2757	2.003
K	1.956	2.6345	1.1266	1.8978
Mg	1.6983	1.2253	1.566	1.6065
Na	1.1572	1.5743	1.193	0.9128
Mn	0.697	0.1771	0.2259	0.8764
Sr	0.6074	0.6114	0.5266	0.659
Cl	0.5115	0.988	0.5811	0.5843
P	0.4614	0.2842	0.5446	0.3421
As	0.2913	0.3662	0.6202	0.4907

Table S2. ncRNA statistics

Non-coding RNA type	Copy number	Average length	Total length	Genome proportion
tRNA	536	76.54	41,027	0.0599%
rRNA	25	2,269.44	56,736	0.0828%
sRNA	45	59.48	2,677	0.0039%
snRNA	101	74.89	7,564	0.011%
miRNA	1,915	54.18	103,764	0.1514%

Table S3 *S. himartioides* DTW genome database annotation statistical results

Database	Number and proportion of gene annotations	Database	Number and proportion of gene annotations
P450	3,083 (16.78%)	CWDE	46 (0.25%)
TF	525 (2.85%)	NR	13,647 (74.27%)
CAZy	500 (2.72%)	GO	7,266 (39.54%)
IPR	10,821 (58.89%)	KEGG	4,455 (24.24%)
KINASE	174 (0.94%)	DBCAN	426 (2.31%)
SWISSPROT	2,638 (14.35%)	TCDB	380 (2.06%)
NOG	8,634 (46.99%)	PHOSPHATASE	46 (0.25%)
CARD	3 (0.01%)	KOG	2,082 (11.33%)

Table S4. Fungistatic agents used in the experiment

Fungistatic agents	Main components	ppm (m/v)
K100	0.75% Isothiazole ketone	0.5%
BC01	3% Isothiazole ketone	0.05%, 0.3%
BC08	quaternary ammonium salt, 1.02 g/mL	0.5%, 3.5%
BC14	14% Isothiazole ketone	0.025%, 0.5%
